# Impaired Activity of Ryanodine Receptors Contributes to Calcium Mishandling in Cardiomyocytes of Metabolic Syndrome Rats

**DOI:** 10.3389/fphys.2019.00520

**Published:** 2019-04-30

**Authors:** Gaudencio Fernández-Miranda, Tatiana Romero-Garcia, Tarín P. Barrera-Lechuga, Martha Mercado-Morales, Angélica Rueda

**Affiliations:** Departamento de Bioquímica, Centro de Investigación y de Estudios Avanzados del IPN (CINVESTAV), Mexico City, Mexico

**Keywords:** ryanodine receptor, calcium sparks, ryanodine binding, SERCA pump, cardiomyocytes, calcium mishandling, metabolic syndrome

## Abstract

Metabolic syndrome (MetS) has become a global epidemic. MetS is a serious health problem because of its related cardiovascular complications, which include hypertension and delayed heart rate recovery after exercise. The molecular bases of cardiac dysfunction in MetS are still under scrutiny and may be related to anomalies in the activity and expression of key proteins involved in the cardiac excitation–contraction coupling (ECC). The cardiac Ca^2+^ channel/ryanodine receptor (RyR2) participates in releasing Ca^2+^ from internal stores and plays a key role in the modulation of ECC. We examined alterations in expression, phosphorylation status, Ca^2+^ sensitivity, and *in situ* function (by measuring Ca^2+^ sparks and Ca^2+^ transients) of RyR2; alterations in these characteristics could help to explain the Ca^2+^ handling disturbances in MetS cardiomyocytes. MetS was induced in rats by adding commercially refined sugar (30% sucrose) to their drinking water for 24 weeks. Cardiomyocytes of MetS rats displayed decreased Ca^2+^ transient amplitude and cell contractility at all stimulation frequencies. Quiescent MetS cardiomyocytes showed a decrease in Ca^2+^ spark frequency, amplitude, and spark-mediated Ca^2+^ leak. The [^3^H]-ryanodine binding data showed that functionally active RyRs are significantly diminished in MetS heart microsomes; and exhibited rapid Ca^2+^-induced inactivation. The phosphorylation of corresponding Ser2814 (a preferential target for CaMKII) of the hRyR2 was significantly diminished. RyR2 protein expression and Ser2808 phosphorylation level were both unchanged. Further, we demonstrated that cardiomyocyte Ca^2+^ mishandling was associated with reduced SERCA pump activity due to decreased Thr17-PLN phosphorylation, suggesting a downregulation of CaMKII in MetS hearts, though the SR Ca^2+^ load remained unchanged. The reduction in the phosphorylation level of RyR2 at Ser2814 decreases RyR2 availability for activation during ECC. In conclusion, the impaired *in situ* activity of RyR2 may also account for the poor overall cardiac outcome reported in MetS patients; hence, the SERCA pump and RyR2 are both attractive potential targets for future therapies.

## Introduction

Metabolic syndrome (MetS) is a cluster of biochemical and physiological risk factors for cardiovascular disease and diabetes mellitus type 2 (DM2); it represents a severe public health problem around the world (Alberti et al., 2009). Risk factors for MetS include obesity (particularly central obesity), elevated triglyceride (TG) levels, low high-density lipoprotein cholesterol (HDL-C) levels, high blood pressure, and dysglycemia. Insulin resistance is considered to be the critical factor underlying MetS, though the pathogenesis remains unclear (Alberti et al., 2009). The definition of what clinically constitutes MetS has generated considerable debate; however, it is generally accepted that a combination of at least three or more of the risk factors must exist to diagnose MetS (Nolan et al., 2017; Tune et al., 2017). People diagnosed with MetS exhibit delayed heart rate recovery after exercise and 50–60% higher cardiovascular risk than those without it (Sung et al., 2006; Qiao et al., 2007). Specifically, MetS is associated with a twofold increase in cardiovascular mortality, myocardial infarction, and stroke (Mottillo et al., 2010). MetS-associated alterations in heart function include impaired myocardial contractility and diastolic dysfunction that predisposes to congestive heart failure (HF) (Tune et al., 2017). At the molecular level in cardiac cells, excitation–contraction coupling (ECC) initiates with T-tubule membrane depolarization, which induces L-type Ca^2+^ channel (LTCC) activation. Ca^2+^ entry is not of sufficient magnitude to activate the contractile machinery but triggers a larger Ca^2+^ release from the stores in the sarcoplasmic reticulum (SR) via the activation of the intracellular Ca^2+^ channel/ryanodine receptor (RyR2), thus promoting cell contraction. This process is known as Ca^2+^-induced Ca^2+^ release (CICR). Relaxation takes place after Ca^2+^ removal, primarily through its recapture into the intracellular Ca^2+^ stores by the sarco/endoplasmic reticulum Ca^2+^ ATPase (SERCA pump) and its extrusion by the action of both the Na^+^/Ca^2+^ exchanger (NCX1) and the plasma membrane calcium ATPase (PMCA) (Fabiato, 1983; Bers, 2014). ECC defects in MetS cardiomyocytes are chiefly characterized by a slow rate of shortening and relengthening. Moreover, depressed cell shortening and slower cytosolic Ca^2+^ clearing have been documented in cardiomyocytes of the prediabetic sucrose-fed rat model at early stages of MetS development (6–18 weeks of sucrose treatment) (Dutta et al., 2001; Hintz and Ren, 2002; Davidoff et al., 2004; Wold et al., 2005; Vasanji et al., 2006; Balderas-Villalobos et al., 2013; Okatan et al., 2016), but the participation of the RyR2 has not been fully elucidated.

The study of RyR2 dysregulation in cardiovascular diseases—including those of inherited and non-inherited etiology—has gained considerable interest in recent years due to its crucial role in the development of HF and cardiac arrhythmias. In the context of type 2 diabetic cardiomyopathy, depressed RyR2 activity is reportedly linked to a reduction in its protein expression; however, other studies did not observe changes in RyR2 at the protein level (Pereira et al., 2006, 2014). In the context of MetS, the function, expression, and phosphorylation status of cardiac RyR are still under analysis. Moreover, altered RyR phosphorylation status has been linked to the impairment of RyR activity, which leads to diabetic cardiomyopathy (Belke et al., 2004; Pereira et al., 2014). Additionally, intricate effects of both protein kinase A- (PKA) and Ca^2+^/Calmodulin-dependent protein kinase II- (CaMKII) mediated RyR phosphorylation have been reported in cardiomyocytes from prediabetic rats, resulting in defective RyR2 regulation (Okatan et al., 2016; Sommese et al., 2016).

Alterations in the *in situ* activity, expression, and regulation by phosphorylation of RyR2 have not been thoroughly evaluated in rat experimental models of MetS, particularly after 24-week treatment with 30% sucrose in drinking water, which elicits a condition resembling a chronic state of MetS in humans. Thus, we sought to examine RyR2 performance in the sucrose-induced MetS rat model. We evaluated the *in situ* activity of RyRs in cardiomyocytes by characterizing electrically-stimulated Ca^2+^ transients, Ca^2+^ spark properties, and spark-mediated Ca^2+^ leak. We also used biochemical approaches ([^3^H]-Ryanodine binding and Western Blots assays) to quantify functional RyR2 and to measure the Ca^2+^ sensitivity, protein expression, and phosphorylation status at Ser2808 and Ser2814 of RyR2 in heart homogenates and SR-enriched membranes.

## Materials and Methods

### Development of the Sucrose-Induced Metabolic Syndrome Model

Male *Wistar* rats, aged 25 days, were divided into two groups and maintained under a dark–light cycle of 12 h and controlled temperature of 22 ± 2°C. The first experimental group (MetS) received 30% sucrose (refined commercial sugar) in their drinking water and commercial rat chow (PicoLab Rodent Diet 20, LabDiet, St. Louis, MO, United States) *ad libitum* for 24 weeks. The second group (control, C) received water and commercial rat chow *ad libitum* during the same 24 weeks. Measurement of systolic blood pressure (SBP) was carried out by the tail-cuff method with a sensor connected to a pressure transducer and a PC equipped with special software (Grass PolyView) for data capture and processing. Recordings were taken in quadruplicate and were compared with those from invasive techniques: no difference has been found, in agreement with previous reports (Perez-Torres et al., 2009; de Alba-Aguayo et al., 2017).

### Measurement of Serum Glucose, Triglycerides, Total Cholesterol, and HDL-Cholesterol

After fasting overnight, rats were treated with heparin (1000 units/kg) and anesthetized with sodium pentobarbital (50 mg/kg, i.p.). Anesthetized animals were subjected to a thoracotomy, during which the heart was exposed and excised rapidly, and blood samples were collected immediately from the sectioned aorta. The serum levels of glucose, triglycerides, total cholesterol, and HDL-cholesterol were measured using glucose and lipid panel strips with the CardioCheck PA analyzer (PTS Diagnostics, Indianapolis, IN, United States).

### Cardiomyocyte Isolation

Left ventricle myocytes were enzymatically isolated from MetS and control rat hearts following a previously reported protocol (de Alba-Aguayo et al., 2017). Animals were treated with heparin (1000 units/kg) and anesthetized with sodium pentobarbital (50 mg/kg, i.p.). The heart was excised rapidly via a thoracotomy and placed in ice-cold (0°C) oxygenated Tyrode solution containing (in mM): NaCl 130, KCl 5.4, NaH_2_PO_4_ 0.4, MgCl_2_ 0.5, glucose 22, Hepes 25 and insulin 10^–3^ (pH 7.4 with NaOH). The aorta was cannulated above the aortic valve and heart was perfused by gravity with warm (37°C) Tyrode solution supplemented with 0.1 mM EGTA for 5 min. Enzyme solution containing 0.8 g/L collagenase Type II (Worthington Biochemical, Corp., Lakewood, NJ, United States) in Tyrode solution supplemented with 0.1 mM CaCl_2_ was then perfused until the aortic valve was digested (confirmed by the increased outflow of perfusate). The heart was transferred to a Petri dish containing enzyme solution supplemented with 1 mg/mL bovine serum albumin (BSA) and gently shaken for 2–3 min at 37°C to disperse individual myocytes. The resulting cell suspension was centrifuged for 3 min at 170 × *g*. The cell pellet was suspended in Tyrode solution supplemented with 0.5 mM CaCl_2_ and was centrifuged again at the same speed. Finally, the cell pellet was suspended in storage solution containing Tyrode solution supplemented with 1 mM CaCl_2_.

### Recording of Ca^2+^ Sparks, Ca^2+^ Transients, and Assessment of SR Ca^2+^ Load

Spontaneous Ca^2+^ sparks and field-stimulated Ca^2+^ transients were imaged in Fluo-3 loaded cardiomyocytes as previously reported (de Alba-Aguayo et al., 2017), with some modifications. Cells were superfused with recording solution (in mM: NaCl 130, CaCl_2_ 1.8, MgCl_2_ 2.0, KCl 5.4, glucose 10, HEPES 10, insulin 0.01, pH 7.4) and 2D-images were obtained with a confocal microscope (Leica TCS SP5, Leica Microsystems, Wetzlar, Germany) in the line-scan mode (1.9 ms/line). To obtain electrically-stimulated Ca^2+^ transients, cells were paced at different frequencies using a Grass stimulator set at 70 V and pulse length of 20 ms; 5 images of the cell were taken for each stimulation frequency. The Y-axis of the image indicates the length of the cell (in μm) and the X-axis indicates the scan time (in s). Fluo-3 was excited at 488 nm using an argon laser at 2% intensity. The emitted fluorescence of the dye was measured at 510 nm. To determined SR Ca^2+^ load, myocytes were paced at 1 Hz for at least 1 min to restore steady state SR Ca^2+^ load, then confocal laser scanning was initiated and four electrically-evoked Ca^2+^ transients were followed by rapid application of caffeine (10 mM) (Fernández-Velasco et al., 2009; de Alba-Aguayo et al., 2017).

### Analysis of Confocal Images

Image analysis was performed using IDL 5.5 software (Research Systems, Inc.) running a custom protocol written by Ana Maria Gómez (Inserm UMR-S 1180, Châtenay-Malabry, France), including baseline fluorescence correction and normalizing fluorescence levels (F) with respect to basal fluorescence (F_0_) (de Alba-Aguayo et al., 2017). The fluorescence transient was obtained by averaging the fluorescence values in a 1.4-μm frame over time. Amplitude was measured as the maximum value of F/F_0_, where F is the fluorescence signal, and F_0_ is the basal fluorescence (measured as the average of the 50 lowest values on the fluorescence transient). Cell shortening was reported in % respect to total cell length. Cells with cell shortening values below 4.5% were not considered in the analysis. Decay Time (in ms) was determined at 50% of recovery of the Ca^2+^ transient peak. Parameters such as amplitude (F/F_0_), frequency (events/s*100 μm), full duration at half maximum (FDHM in ms), full width at half maximum (FWHM in μm), time-to-peak (in ms) and decay time constant (in ms), were analyzed for Ca^2+^ sparks (de Alba-Aguayo et al., 2017). The spark-mediated Ca^2+^ leak was calculated according to the method reported by Biesmans and collaborators, in which the spark-mediated Ca^2+^ leak is defined as spark frequency*spark mass; and spark mass is calculated as spark amplitude*duration*width (Biesmans et al., 2011).

### Preparation of Heart Homogenates and SR-Enriched Fractions

Heart homogenates and SR-enriched fractions were prepared from left-ventricle tissue pulverized to a fine powder with a mortar and pestle over liquid N_2_. Tissue powder was suspended in homogenization buffer (in mM: sucrose 300, NaF 20, HEPES 20, aprotinin 5.2 × 10^4^, benzamidine 0.5, leupeptin, 0.012, PMSF 0.1; pH 7.2 with KOH) and homogenized with a Potter-Elvehjem homogenizer (Cole-Parmer, Vernon Hills, IL, United States) and spun at 2000 × *g* for 10 min; the resulting supernatant (homogenate) was spun at 8000 × *g* for 10 min; SR-enriched fractions (or microsomes) were isolated using the second supernatant by ultracentrifugation at 40000 × *g* for 30 min at 4°C. The resulting pellets were suspended in homogenization buffer and protein concentration was determined using the Lowry method as previously reported (de Alba-Aguayo et al., 2017).

### Measurement of SR Ca^2+^ Uptake

Fura-4F acid form (pentapotassium salt, Molecular Probes/Thermo Fisher Scientific, Inc.) was added outside SR-membrane vesicles and used to monitor the SERCA-dependent Ca^2+^ uptake accordingly to a previously described method (de Alba-Aguayo et al., 2017). SR Ca^2+^ uptake was measured in a buffer containing (in mM): KCl 100, MgCl_2_ 4, MOPS 20, potassium oxalate 10, ATP-Mg 1.25, creatine phosphate 1.5, pH 7.4 with KOH, and 0.3 U/ml creatine phosphokinase. 10 μM Ruthenium Red was added to inhibit Ca^2+^ release through RyRs. Fura-4F was added to a final concentration of 0.5 μM. 600 μg of protein from SR-enriched vesicles were added to a cuvette with uptake buffer and equilibrated at 37°C during 5 min with stirring. Fluorescence was measured in a QM-8 spectrofluorometer (*PTI*, excitation wavelengths at 340, 360, and 380 nm; emission was collected at 510 nm). The addition of 5 μM CaCl_2_ initiated Ca^2+^ uptake reactions; the fluorescence was recorded up to 900 s. The experiment was finished by adding Thapsigargin (1 μM). To determine R_max_, 200 μM CaCl_2_ was added; to measure R_min_, 250 μM EGTA was added. Ratio values of fluorescence (F340/F380) were subsequently transformed into Ca^2+^ concentrations using the Grynkiewicz equation (Grynkiewicz et al., 1985). Ca^2+^ uptake speed was calculated as the first derivative of the monoexponential decay equation used to fit the Ca^2+^ uptake curve.

### [^3^H]-Ryanodine Binding Assays

[^3^H]-ryanodine binding experiments were performed as previously described using SR-enriched fractions (Fernández-Velasco et al., 2009; de Alba-Aguayo et al., 2017). [^3^H]-ryanodine saturation curves were carried out in medium containing 1 M KCl, 0.1 mM CaCl_2_, 20 mM HEPES (pH 7.2 with KOH); and [^3^H]-ryanodine in the range of 0.625 to 20 nM. Equilibrium binding data were fitted to the one-site model (Hill equation), apparent dissociation constant (*Kd*) and total functional RyRs (*Bmax*) were calculated from the fitted curve. The Ca^2+^ dependence of [^3^H]-ryanodine binding was assessed in incubation medium containing 0.2 mM KCl, 20 HEPES (pH 7.2 with KOH), 50 μg of SR-enriched fractions, 10 nM [^3^H]-ryanodine, 1 mM EGTA, and CaCl_2_ necessary to set free Ca^2+^ in the range of 10 nM to 10 mM (total volume 100 μl). Ca^2+^-EGTA ratios were calculated using the MaxChelator program^1^ (Bers et al., 2010). All incubations lasted 90 min at 36°C. Samples were run in duplicate, filtered onto glass fiber filters (Whatman GF/B), and washed three times with 5 ml of cold water using a Brandel M-24R cell harvester. The filters were placed in scintillation vials, 8 ml of liquid scintillation mixture was added, and the retained radioactivity was measured in a Beckman LS-6500 β-counter. The specific binding was defined as the difference between the binding in the absence (total binding) and presence (non-specific binding) of 20 μM unlabeled ryanodine. Data represent the normalized mean ± SEM of the indicated *n* in the respective figure. Fitting of data was accomplished with the computer program OriginPro 8 (OriginLab Corporation, Northampton, MA, United States) to the following equation: *B* = *B*_max_([Ca^2+^]^na^/([Ca^2+^]^na^+*K*_a_^na^))(1–[Ca^2+^]^ni^/([Ca^2+^]^ni^+*K*_i_^ni^))+*C*, modified from Meissner’s group (Liu et al., 1998), where *B* is the [^3^H]-ryanodine binding value at a given [Ca^2+^], *B_max_* is the binding maximum, *K_a_* and *K_i_* are Hill activation and inactivation constants, respectively, *n_a_* and *n_i_* are the respective Hill coefficients, and *C* is an initial [^3^H]-ryanodine binding value at very low [Ca^2+^] (pCa8) (Fernández-Velasco et al., 2009; de Alba-Aguayo et al., 2017).

### SDS-PAGE and Western Blot Analysis

SDS-PAGE and Western Blots were performed as a modification of previously reported protocols (Fernández-Velasco et al., 2009; de Alba-Aguayo et al., 2017). Gradient polyacrylamide gels (4–16%). Gels were loaded with the indicated μg of protein from heart homogenates in Laemmli buffer. Each gel was transferred onto a PVDF membrane for 2 h, 100 V at 4°C in a humid chamber. PVDF membrane was blocked from non-specific binding with 5% non-fat dry milk in PBS-T buffer (composition in mM: KH_2_PO_4_ 2.9, Na_2_HPO_4_ 10.07, NaCl_2_ 150.58 and Tween 20, 0.1%) for 1 h, before incubation with primary antibody against RyR2 (C3-33 dil: 1:5000, Cat# MA3-916, Thermo Fisher, Inc., Waltham, MA United States), pSer2808RyR2 (dil: 1:5000, Cat# A010-30, Badrilla, Leeds, United Kingdom), pSer2814RyR2 (dil: 1:5,000 Cat# A010-31, Badrilla, Leeds, United Kingdom), SERCA2a (dil: 1,5000 Cat# A010-20, Badrilla, Leeds, United Kingdom), PLN (dil: 1:25,000 Cat# A010-14, Badrilla, Leeds, United Kingdom), pSer16PLN (dil: 1:3,000 Cat# A010-12, Badrilla, Leeds, United Kingdom), pThr17PLN (dil: 1:5,000 Cat# A010-13, Badrilla, Leeds, United Kingdom), CaMKII (dil: 1:2,500 Cat# 4436S, Cell Signaling, Danvers, MA, United States), and GAPDH (dil: 1:100,000 Cat# AM4300, Ambion^®^, Thermo Fisher Scientific, Waltham, MA, United States) for 2 h at room temperature. After washing, the membranes were incubated with corresponding secondary peroxidase-conjugated antibodies (dil 1:5,000 of goat anti-mouse IgG peroxidase conjugated, Cat. No. 401215, or goat anti-rabbit IgG peroxidase conjugated, Cat. No. 401315; both from Calbiochem^®^ Merck, Co., Kenilworth, NJ, United States) for 1 h. The membranes were washed (3X, 10 min each with PBS-T buffer). Proteins were visualized by chemiluminescent reaction (SuperSignal^®^ West Pico Chemiluminescent Substrate, Thermo Fisher Scientific, Waltham, MA, United States) and the relative amount of protein was determined by densitometric analysis using Kodak MI SE Software (v.5.01.30. Molecular Imaging Software).

### Statistical Data Analysis

Data are presented as the mean ± SEM of indicated independent determinations, where “N” was used to designate the number of animals or hearts; and “n” to specify the number of cells, Ca^2+^ release events, or experiments. Statistical significance was evaluated by Student’s *t*-test or one-way ANOVA followed by Tukey *post hoc* test, when appropriated (OriginPro 8 software from Origin Lab Corporation, Northampton, MA, United States). Significance was defined at *P* < 0.05.

## Results

### Characteristics of the Chronic Sucrose-Induced MetS Model

Male rats that received 30% sucrose in the drinking water for 24 weeks developed several of the hallmark characteristics of MetS: for instance, a significant increase in body weight (32%) and visceral fat accumulation (2.4-fold) compared to the control group (Table 1). Additionally, average SBP was significantly higher in MetS rats than controls (Table 1). Although the heart weight was slightly augmented in the MetS condition, features such as heart weight-to-body weight ratio, left ventricle weight, and left ventricle-to-heart weight ratio showed no alterations, indicating the lack of severe structural remodeling in the heart (Table 1). Serum parameters evidenced the development of hypertriglyceridemia (2.6-fold increase in serum triglycerides levels); and a significant increase in the TG-to-HDL-C ratio, which is indicative of a higher degree of cardio-metabolic risk in the insulin-resistant condition (Salazar et al., 2014). These alterations in both body and serum parameters are strong evidence of the development of MetS in rats as a consequence of the sucrose-rich diet (Wong et al., 2016).

**TABLE 1:** Body characteristics and biochemical parameters of metabolic syndrome and control animals.

Body parameters	Control	Metabolic syndrome
Body weight (g)	545.0±27.9	$721.1\pm 33.9^{**}$
Visceral fat (g)	9.8±1.1	$23.9\pm 1.5^{**}$
Systolic pressure (mmHg)	113.8±1.7	$143.2\pm 4.9^{**}$
Heart weight (g)	1.8±0.1	$2.0\pm 0.1^{*}$
Hw/Bw ratio × 100	0.34±0.02	0.35±0.03
Left ventricle weight (g)	1.1±0.05	1.1±0.05
LV/Hw ratio	0.63±0.03	0.54±0.05
**Biochemical parameters**		
Glucose (mg/dL)	71.4±3.6	77.0±4.4
Total cholesterol (mg/dL)	72.9±14.5	65.4±6.9
HDL-C (mg/dL)	41.3±5.5	35.8±3.0
Triglycerides (mg/dL)	84.6±12.0	$219.6\pm 20.5^{**}$
TG/HDL-C ratio	1.93±0.45	$6.13\pm 0.69^{**}$

Data are means ± SEM of *N* = 14 control rats and *N* = 12 MetS rats. Bw, body weight; HDL-C, high-density lipoprotein cholesterol; Hw, heart weight; LV, left ventricle; TGs, triglycerides. *P < 0.05 and **P < 0.001 vs. Control group.

### Diminished Ca^2+^ Transient Amplitude in Cardiomyocytes of Metabolic Syndrome Rats

**FIGURE 1:**
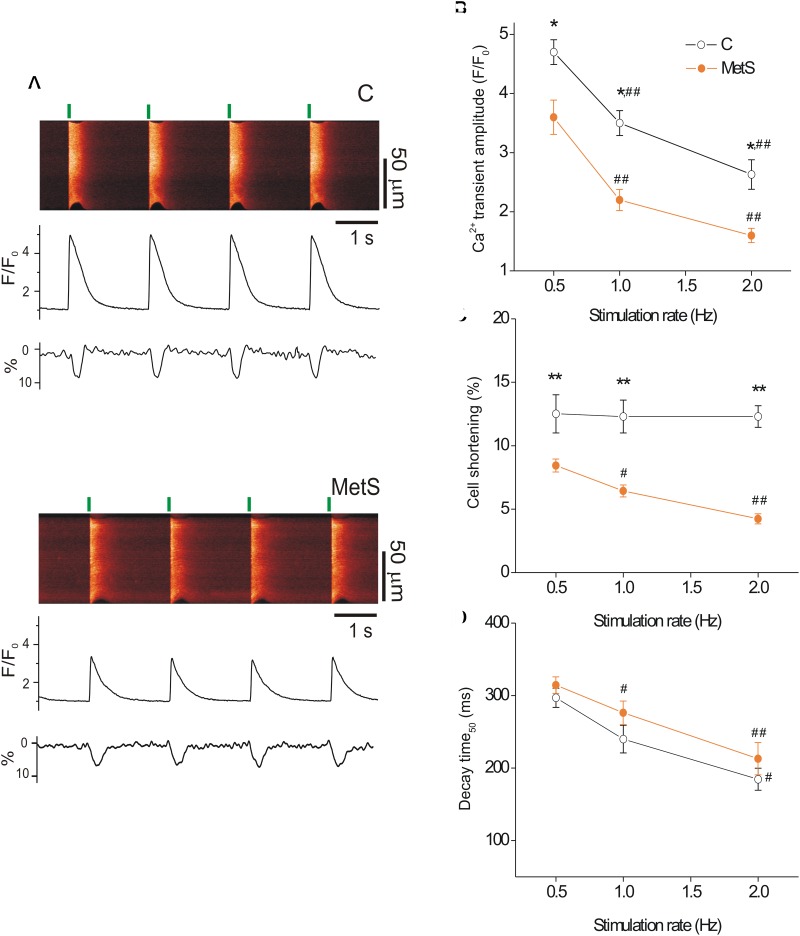
Metabolic syndrome (MetS) cardiomyocytes exhibit a rate-dependent decrease in Ca^2+^ transient amplitude and contraction. Representative line-scan images **(A)** recorded during electric stimulation (0.5 Hz) of Fluo 3-loaded ventricular myocytes isolated from control (C) and MetS rats. Green lines indicate the start of electric stimulation. The corresponding normalized fluorescence traces (F/F_0_) and contraction profiles (in %) are shown below. **(B)** Average of Ca^2+^ transient amplitude (expressed as F/F_0_, where F is the peak fluorescence signal and F_0_ the diastolic fluorescence) of control (*open circles*) and MetS (*orange circles*) cardiomyocytes obtained by field stimulation at 0.5, 1, and 2 Hz. Average cell shortening **(C)**, and decay time at 50% of the normalized Ca^2+^ transient **(D)** at 0.5, 1, and 2 Hz in *n* = 17 control cells vs. *n* = 32 MetS cardiomyocytes. *P < 0.05, **P < 0.001 respect to control cells. ^#^*P* < 0.05, ^##^*P* < 0.001 respect to 0.5 Hz in the same condition.

Previous studies have demonstrated depressed cardiac contractile function in sucrose-fed animals, evidenced by the reduction of ejection fraction, left ventricle systolic function, and heart fractional shortening (Vasanji et al., 2006). Moreover, reduced peak tension in papillary muscle strips has been reported in MetS condition (Okatan et al., 2016). Because the intensity of cardiomyocyte contractile response relies on the magnitude and duration of the intracellular Ca^2+^ transient (Spurgeon et al., 1992), we investigated whether MetS affected electrically-evoked Ca^2+^ transients and associated cell shortening in single cardiomyocytes at three different stimulation frequencies. Figure 1A shows representative confocal images (acquired in *line scan* mode) of Fluo-3-loaded cardiomyocytes from control (*top*) and MetS (*bottom*) rats at 0.5 Hz, with their corresponding Ca^2+^ transient profiles (F/F_0_), cellular shortening (% of resting cell size), and Ca^2+^ transient decay time (ms). Cardiomyocytes of both experimental groups exhibited a rate-dependent fall in the Ca^2+^ transient amplitude; however, in the MetS condition, the magnitude of the Ca^2+^ transient was consistently lower than in control cells (Figure 1B). In MetS cells, the amplitude of the Ca^2+^ transient represented 76.6, 62.8, and 61.5%, of corresponding Ca^2+^ transient amplitude in control cells, at 0.5, 1, and 2 Hz, respectively. Interestingly, a rate-dependent decline in cell contraction was found in the MetS group, with a significant reduction of 32.8, 48, and 65.9% in cell shortening of MetS cardiomyocytes at 0.5, 1, and 2 Hz, respectively (Figure 1C). We investigated whether alterations in the distance between T-tubules in cardiomyocytes to estimate sarcomere length (SL), could account for these results (see Supplementary Materials and Methods), since SL above or below the optimal range is known to contribute to a decrease in cardiomyocyte contractility. However, we found similar T-tubule distances in RyR-immunostained cardiomyocytes from both experimental groups (average T-tubule distance, in μm: 1.67 ± 0.03 in *n* = 5 control cells, vs. 1.64 ± 0.019 in *n* = 5 MetS cells, *P* = 0.535. Supplementary Figure 1), ruling out the presence of structural T-tubule remodeling in MetS cardiomyocytes. Analysis of Ca^2+^ transient kinetics revealed that the decay time (at 50% of the peak) was slightly increased in MetS cells at all frequencies (Figure 1D), suggesting that the Ca^2+^ clearing mechanisms are slowed. The Ca^2+^ handling abnormalities could be related to a decreased rate of SR Ca^2+^ uptake and/or an increased SR Ca^2+^ leak; we therefore evaluated the Ca^2+^ spark-mediated Ca^2+^ leak.

### Reduced Diastolic Ca^2+^ Leak in Cardiomyocytes of Metabolic Syndrome Rats

**FIGURE 2:**
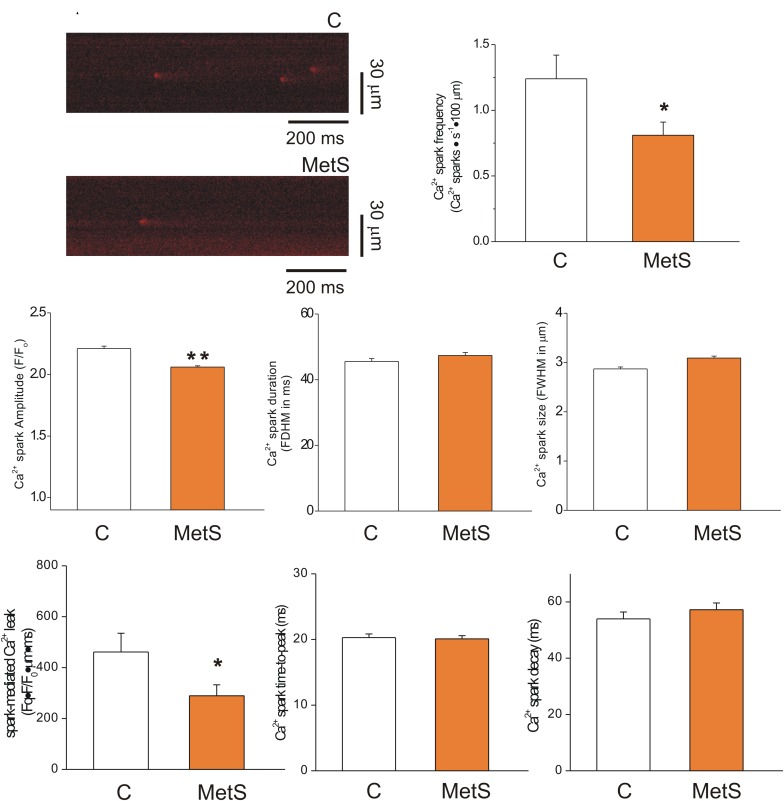
Reduced spark-mediated Ca^2+^ leak in cardiomyocytes of MetS animals. Representative line-scan images (2 ms/line) of spontaneous Ca^2+^ sparks recorded in Fluo 3-loaded cardiomyocytes from control (C) and MetS hearts **(A)**. Bar graphs comparing the average frequency (**B**, events/s⋅100 μm), amplitude (**C**, F/F_0_), duration (**D**, FDHM in ms), size (**E**, FWHM in μm), spark-mediated Ca^2+^ leak (**F**, reported as spark frequency*spark mass), time-to-peak (**G,** in ms), and decay time (**H,** in ms) of Ca^2+^ sparks in control (*white bars*; *n* = 907 events recorded in 41 cells) and MetS (*orange bars*; *n* = 1050 events recorded in 50 cells) cardiomyocytes. *P < 0.05; **P < 0.001 respect to control parameters.

The extent of SR Ca^2+^ leak impacts the SR Ca^2+^ available for release, causing systolic dysfunction. Increased SR Ca^2+^ leak elevates diastolic Ca^2+^ level, contributing to diastolic dysfunction; causes triggered arrhythmias and it is energetically costly (Bers, 2014). Thus, in a first approach, we analyzed the Ca^2+^ -spark mediated Ca^2+^ leak in intact Fluo 3-loaded cardiomyocytes of both experimental groups. Figure 2A shows representative confocal images of spontaneous Ca^2+^ sparks taken from control (*top*) and MetS (*bottom*) cardiomyocytes in *line scan* mode. Analyses of Ca^2+^ spark characteristics are summarized in Figures 2B through 2H. Ca^2+^ sparks recorded in MetS cells showed significantly reduced frequency (by 34.7%, Figure 2B) and amplitude (by 7%, Figure 2C) compared to control cells. Ca^2+^ spark mass calculated as the product of Ca^2+^ spark amplitude*duration*width was lower in MetS cell than in controls, resulting in a significant reduction of the Ca^2+^ spark-mediated Ca^2+^ leak (Figure 2F). Other Ca^2+^ spark parameters such as duration (Figure 2D), size (Figure 2E) time-to-peak (Figure 2G), and decay time (Figure 2H) remained unchanged.

### SR Ca^2+^ Load Is Unaffected in MetS Cardiomyocytes

**FIGURE 3:**
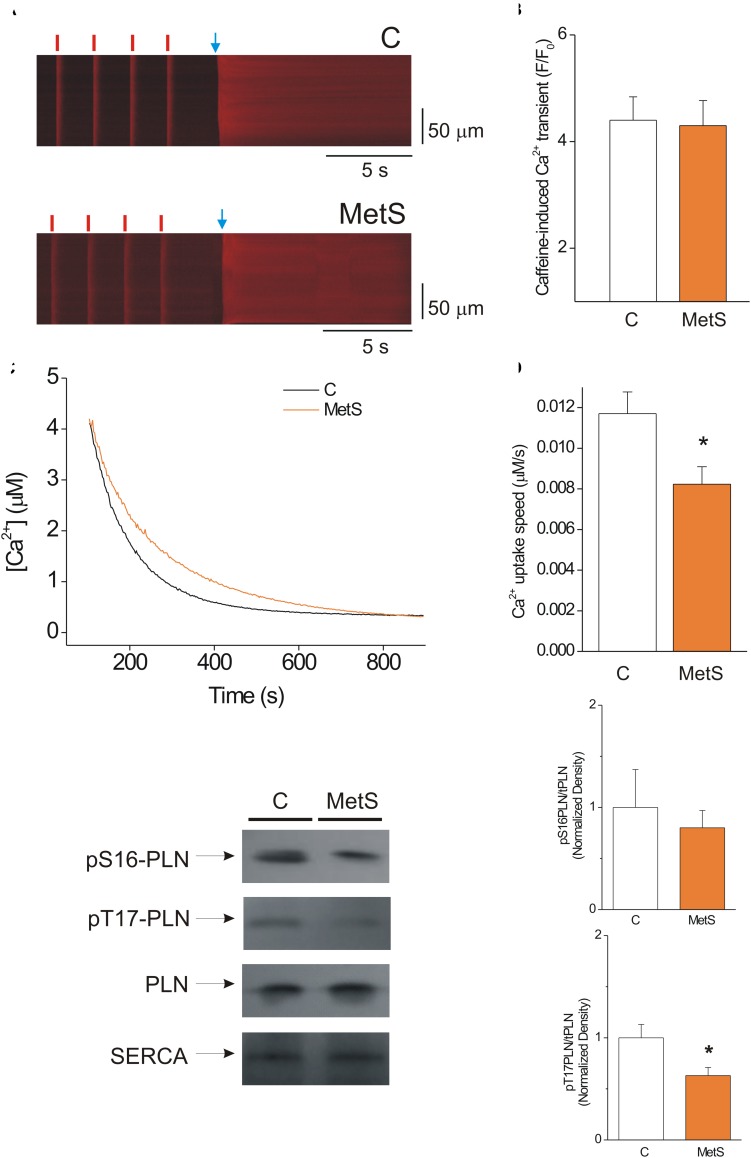
SERCA pump activity is impaired, but this condition does not alter the SR Ca^2+^ load in MetS cardiomyocytes. **(A)** Representative line-scan images of caffeine-induced Ca^2+^ transients recorded in isolated Fluo 3-loaded cardiomyocytes from control (C, *top*) and MetS (*bottom*) rats to determine SR Ca^2+^ loading. Cells were superfused with recording solution and paced at a frequency of 1 Hz (*red lines*) before caffeine challenge (indicated by the *blue arrow*). **(B)** The bar graph represents average values of the caffeine-induced Ca^2+^ transient amplitude (F/F_0_) in control (*white bar*, *n* = 14 cells) and MetS (*orange bar*, *n* = 22 cells) cardiomyocytes. **(C)** Representative traces of Ca^2+^ uptake assays in SR-enriched microsomes obtained from control (C, *black trace*) and MetS (*orange trace*) hearts. **(D)** The bar graph compares the average Ca^2+^ uptake speed (in μM/s) in control (*white bar*, *n* = 10), and MetS (*orange bars, n* = 8) microsomal preparations. **(E)** Representative Western Blot images of phosphorylated PLN at Ser16, Thr17, total PLN, and SERCA pump in whole heart homogenates of control (C) and MetS rats. Bar graphs of normalized phosphorylation levels of pS16 PLN **(F)** and pT17 PLN **(G)** in whole heart homogenates of control (C, *white bars*) and MetS (*orange bars*) rats. *N* = 4–7 heart preparations for each experimental group. All values in graphs are presented as Mean ± SEM. *P < 0.05 vs. control values.

The amount of Ca^2+^ stored inside the SR that is available for release during the cardiomyocyte contraction is primarily determined by the delicate balance between diastolic Ca^2+^ leak and SERCA pump-mediated Ca^2+^ uptake (Zima et al., 2010). Thus, we hypothesized that the decrease in the Ca^2+^ spark-mediated Ca^2+^ leak in MetS cells could be attributable to a significant reduction of the SR Ca^2+^ load. Accordingly, we determined the SR Ca^2+^ load in steady state conditions by measuring the amplitude of the caffeine-induced Ca^2+^ transient in cardiomyocytes previously paced at 1 Hz. Figure 3A shows representative line-scan images of caffeine-induced Ca^2+^ transients recorded in Fluo-3 loaded control and MetS cells. Our results show that the development of MetS did not modify the SR Ca^2+^ loading in steady-state conditions because the amplitude of the caffeine-induced Ca^2+^ transient was similar in both groups (Figure 3B), in agreement with previous reports in sucrose-fed rats at early stages of MetS development (Wold et al., 2005; Balderas-Villalobos et al., 2013). Unchanged SR Ca^2+^ content is incompatible with reduced diastolic Ca^2+^ leak in the MetS cardiomyocytes—unless SERCA pump activity is compromised. Although the decay time of Ca^2+^ transients was found to be slightly increased in MetS cells, this parameter is a combination of several mechanisms, including SR Ca^2+^ uptake, cytoplasmic Ca^2+^ buffering, and plasma membrane Ca^2+^ extrusion, among others (Bode et al., 2011). Therefore, by further analyzing the caffeine-induced Ca^2+^ recordings, we specifically examined SERCA function using a previously reported method (Bode et al., 2011; Delgado et al., 2015). We calculated the SERCA-dependent portion of the rate constant of decay of the Ca^2+^ transient (*k* SERCA) by subtracting the rate constant of decay of the caffeine-evoked Ca^2+^ transient from that of the systolic Ca^2+^ transients. These calculations demonstrated that *k* SERCA was significantly reduced in MetS cardiomyocytes (4.23 ± 0.38 s^–1^, *n* = 6 in control vs. 3.37 ± 0.12 s^–1^, *n* = 8, in MetS group; *P* < 0.05). These data enabled us to hypothesize impaired SERCA pump function in MetS cardiomyocytes.

We therefore used a more direct approach to investigate SERCA pump enzymatic activity by performing a SERCA pump-mediated Ca^2+^ uptake assay in cardiac SR-enriched membranes (Figure 3C). These experiments revealed a 33% decrease in the rate of Ca^2+^ uptake speed in MetS microsomes compared to the control microsomes (Figure 3D). SERCA-pump mediated Ca^2+^ uptake was slowed in MetS microsomes, though SERCA pump protein expression was not affected (normalized SERCA pump expression: 1.0 ± 0.11 in *n* = 11 control samples vs. 1.05 ± 0.14 in *n* = 11 MetS samples; *P* > 0.05, N.S.). This result agrees with our finding of unchanged SR Ca^2+^ load despite the reduced diastolic Ca^2+^ leak observed in MetS cardiomyocytes.

Because PLN regulates SERCA-mediated Ca^2+^ uptake, changes in PLN expression level and/or phosphorylation status would be expected modify SERCA activity (Mattiazzi and Kranias, 2014). To gain insight into the molecular mechanisms involved in the observed SERCA pump dysfunction, we analyzed PLN expression and phosphorylation status of phosphosites Ser16 (a PKA site) and Thr17 (a CaMKII site; Figure 3E). Total PLN expression and PLN/SERCA pump ratio were similar in both experimental conditions (PLN/SERCA pump, normalized data: 1.0 ± 0.16 in *n* = 7 control samples vs. 1.31 ± 0.18 in *n* = 7 MetS samples, *P* < 0.2307). Western Blot data showed a slight but not significant decrease in the phosphorylation level of Ser16-PLN (Figure 3F). However, Thr17-PLN phosphorylation was significantly reduced in MetS heart homogenates (Figure 3G), which correlates with the reduction in SERCA activity observed in SR Ca^2+^ uptake assays (Figures 3C,D).

### Dysfunctional Cardiac RyRs in the Metabolic Syndrome Condition

**FIGURE 4:**
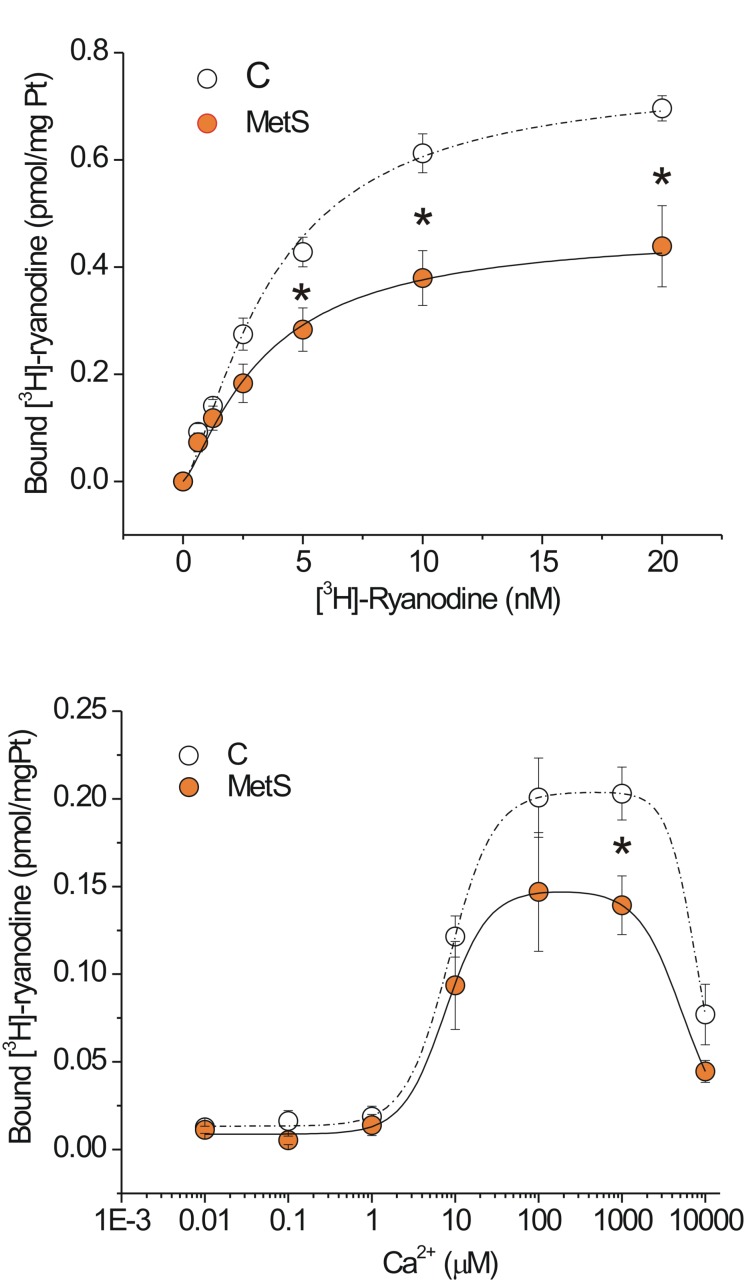
Functional ryanodine receptors are decreased and showed rapid Ca^2+^-induced inactivation in MetS hearts. Saturation binding curve with [^3^H]-ryanodine. **(A)** Aliquots of 50 μg of protein from microsomes (SR-enriched fraction) from control (*N* = 6) and MetS (*N* = 5) hearts were incubated with [^3^H]-ryanodine (concentration range of 0.625–20 nM) in incubation medium (containing in mM: 1000 KCl, 0.1 CaCl_2_ 20 HEPES, pH 7.2) for 90 min at 37°C. **(B)** Specific Ca^2+^-dependent [^3^H]-ryanodine binding curves of SR-enriched fractions from control (*open symbols; N* = 9) and MetS (*orange symbols*; *N* = 6) hearts. Microsomes were incubated with 7 nM [^3^H]-ryanodine and the indicated [Ca^2+^]_i_ at 37°C for 90 min. Values have been normalized and fitted to a modified version of the equation *B* = *B*_max_([Ca^2+^]^na^/([Ca^2+^]^na^+*K*_a_^na^))(1–[Ca^2+^]^ni^/([Ca^2+^]^ni^+*K*_i_^ni^))+*C*. Non-specific [^3^H]-ryanodine binding was determined in the presence of 20 μM ryanodine and subtracted from all reported values. *Open symbols*, control; *orange symbols*, MetS; *dashed line* indicates the fitting curve for control and *dark solid line* the fitting curve for MetS microsomes. *P < 0.05 with respect to corresponding control values.

In our experimental model of MetS, the decrease in both the Ca^2+^ transient amplitude and the Ca^2+^ spark-mediated Ca^2+^ leak in isolated cardiomyocytes cannot be explained by a reduction in the SR Ca^2+^ load; therefore, we explored additional alterations in the RyR that might enable us to understand the changes in its activity. The fact that ryanodine binds only to open RyRs enables estimation of functional RyRs under *in vitro* conditions. Figure 4A shows saturation binding curves for [^3^H]-ryanodine in SR-enriched fractions from MetS and control hearts. SR membranes from MetS hearts bound significantly less [^3^H]-ryanodine (38.2%) than those from control hearts. Importantly, the *Kd* for [^3^H]-ryanodine was found in the nanomolar range and remained unchanged (Table 2), indicating that the apparent ryanodine affinity of RyRs remains similar in both experimental groups. We also examined whether the Ca^2+^-dependent activation of [^3^H]-ryanodine binding might differ between MetS and control microsomes. Figure 4B shows bell-shape-like curves for both control and MetS heart microsomes. Both data sets were fitted with the same equation, indicating that RyRs followed a bimodal Ca^2+^ activation/inactivation response as previously reported (Liu et al., 1998; de Alba-Aguayo et al., 2017). The fitted parameters indicate that Ca^2+^ was equally effective in activating RyRs from control and MetS heart microsomes, since the range for *Ka* value remained similar (Table 3) and in agreement with previous reports (Liu et al., 1998). However, RyRs from MetS hearts exhibited rapid Ca^2+^-induced inactivation (Figure 4B), and showed a significant decrease (26.3%) in the total amount of RyRs capable of binding [^3^H]-ryanodine at optimal [Ca^2+^] (indicated by the *Bmax* value in Table 3), with a slight, but not significant, decrease in the initial quantity of active RyR at very low [Ca^2+^] (indicated by the *C* value in Table 3). Thus, our results show an important reduction of active RyR2 that inactivate faster in MetS microsomes, which could help explain the decrease in both Ca^2+^ transient amplitude and the diastolic Ca^2+^ leak in MetS cardiomyocytes despite the unchanged SR Ca^2+^ load.

**TABLE 2:** Parameters of saturation [^3^H]-ryanodine binding curves in microsomes of MetS and control hearts.

Experimental group	*Bmax* (pmol [^3^H]ryanodine/mg Pt)	*Kd* (nM)	Hill coefficient	*N*
Control	0.76 ± 0.06	3.69 ± 0.63	1.4 ± 0.38	6
MetS	0.47 ± 0.08*	3.41 ± 0.43	1.3 ± 0.23	5

Data are Mean ± SEM of indicated heart preparations for each experimental group. *P < 0.05 vs. control group.

**TABLE 3:** Ca^2+^-dependence of [^3^H]-ryanodine binding in microsomes of MetS and controls hearts.

Condition	*Bmax* (pmol [^3^H]ryanodine/mg Pt)	*Ka* (μM)	*Ki* (mM)	*C* (pmol [^3^H]ryanodine/mg Pt)	*N*
Control	0.19 ± 0.01	8.48 ± 1.05	7.6 ± 0.6	0.013 ± 0.001	9
MetS	0.14 ± 0.08*	7.57 ± 0.86	5.2 ± 1.0	0.009 ± 0.003	6

Values are Mean ± SEM of indicated independent determinations for each experimental group. *Bmax* indicates the maximum number of active RyRs, K${}_{a}$ and K${}_{i}$ are Hill activation and inactivation constants, respectively; and C is an initial [${}^{3}$H]-ryanodine binding value at very low [Ca${}^{2+}$] (pCa8). *N* indicates the number of hearts. *P < 0.05 vs. control group.

**FIGURE 5:**
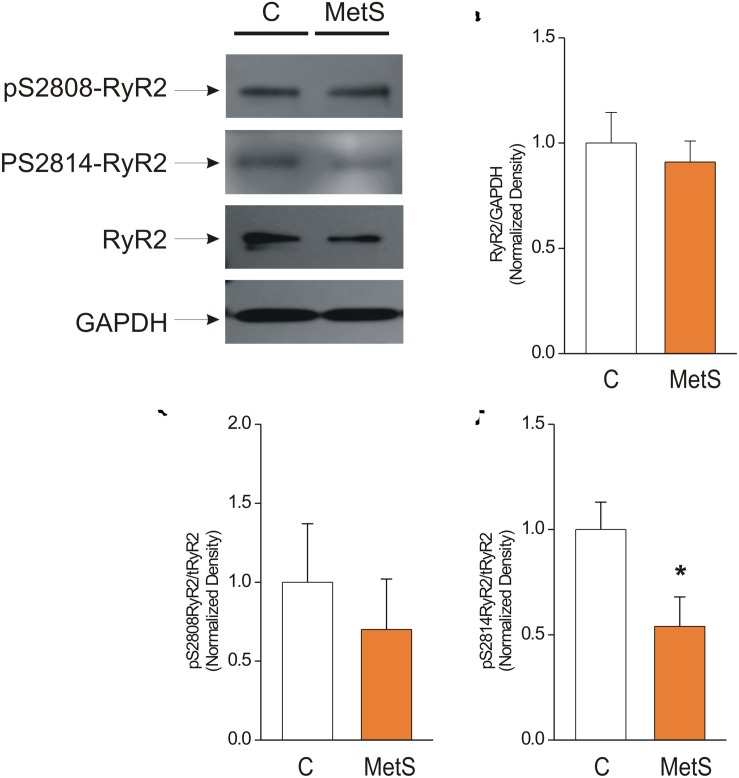
Reduced phosphorylation levels of Cardiac Ryanodine Receptor at Ser-2814 underlie its dysfunctional activity. **(A)** Representative Western Blot images of phosphorylated RyR2 at Ser2808 (pS2808-RyR2), Ser2814 (pS2814-RyR2), and total ryanodine receptor (RyR2) in whole heart homogenates of control (C) and MetS rats. GAPDH signals were used as loading controls. 50 μg of protein from whole heart homogenates of each experimental group (C and MetS) were subjected to 4–16% gradient SDS-PAGE, blotted onto PVDF membranes during 2 h, and probed with the respective primary and secondary antibodies. Bar graphs indicate normalized expression levels of total RyR2 **(B)**, pS2808RyR2/RyR2 total **(C)**, and pS2814RyR2/RyR2 total **(D)** in whole heart homogenates of control (C, *white bars*) and MetS (*orange bars*) rats. *N* = 6–7 heart preparations for each experimental group. All values in graphs are presented as Mean ± SEM. *P < 0.05 vs. control values.

Western blot analysis was used to determine the total amount of RyR2 protein expression in control and MetS heart preparations. We also analyzed the phosphorylation levels of the corresponding Ser2808 (a substrate for several kinases, PKA and CaMKII among them) and Ser2814 (a preferential target for CaMKII) in RyR2, in light of the finding that modifications of RyR phosphorylation level account for the dysfunctional activity of cardiac RyRs in insulin-resistance and prediabetes (Dincer, 2012; Okatan et al., 2016; Sommese et al., 2016). Figure 5A shows representative immunoblots of pSer-2808-RyR2, pSer-2814-RyR2, and total RyR. The bar graph in Figure 5B shows that the total amount of RyR2 was unmodified in MetS heart preparations in comparison with controls (normalized total RyR2 expression: 1.0 ± 0.15 in *N* = 6 control hearts vs. 0.91 ± 0.10 in *N* = 7 MetS hearts; *P* > 0.05, N.S.). Nevertheless, we found a significant reduction (46%) in the phosphorylation status of Ser2814 (Normalized pSer2814RyR2: 1.0 ± 0.13 in *N* = 6 control vs. 0.54 ± 0.14 in *N* = 6 MetS hearts; *P < 0.05. Figure 5D), while the phosphorylation level of Ser2808 remained unchanged (normalized pSer2808RyR2: 1.0 ± 0.37 in *N* = 6 control vs. 0.7 ± 0.32 in *N* = 6 MetS hearts; *P* > 0.05, N.S.; Figure 5C). The decreased levels of pSer2814-RyR2 and pThr17-PLN, both well-known CaMKII target sites, suggests that CaMKII activity might be compromised in our MetS model. Although CaMKII expression was similar in both experimental groups (total CaMKII expression: 1.0 ± 0.07 in *N* = 7 control hearts vs. 1.01 ± 0.07 in *N* = 8 MetS hearts; *P >* 0.05, N.S.; Supplementary Figure 2), we cannot rule out that changes in its activity could account for the Ca^2+^ handling alterations reported in this work. Since CaMKII activity is modulated by a wide range of post-translational modifications, further analysis regarding this issue will be required.

## Discussion

In diabetic cardiomyopathy, the mechanisms that regulate intracellular Ca^2+^ handling are altered and the function of RyR2 is clearly compromised; the latter has been associated with a decrease in RyR2 protein expression and reduced SR Ca^2+^ load (Pereira et al., 2014); however, the data concerning the contribution of RyRs to the Ca^2+^ mishandling in MetS cardiomyocytes has, until now, been lacking.

Thus, the main aim of this study was to more closely examine the *in situ* activity, protein expression, Ca^2+^ sensitivity, and phosphorylation status of the RyR2 in an animal model of MetS. Sucrose administration (30% in drinking water) for 24 weeks successfully generated key features of MetS in rats, characterized by the presence of central obesity, hypertension, hypertriglyceridemia, and increased TG/HDL-C ratio (Table 1). Previous reports in experimental models of MetS at early stages of development induced by administration of either fructose or sucrose in the diet have shown differential development of hypertriglyceridemia, hypertension, and increased abdominal fat accumulation, though not necessarily an increase in body weight (Hintz and Ren, 2002; Davidoff et al., 2004; Schwanke et al., 2006; Balderas-Villalobos et al., 2013; Sommese et al., 2016). Under our experimental conditions, the sucrose-fed rats showed a phenotype closely related with MetS patients, in whom a significant increase in blood presure, TG/HDL-C ratio, body weight, and visceral fat accumulation are considered key cardiovascular risk factors (Wilson et al., 2005). In our MetS model, several hallmark metabolic disturbances are strongly established, leading to a condition that more closely resembles chronic prediabetic state in obese humans. We believe that the differences in our model respect to others are mainly attributable to the prolonged administration of sucrose (e.g., 6 months rather than only 4).

Our results demonstrate the detrimental effect of a high carbohydrate diet on the metabolic condition—and specifically in cardiomyocyte performance, which agrees well with previous publications (Cárdenas et al., 2006; Balderas-Villalobos et al., 2013; Okatan et al., 2016).

Alterations in heart contractile activity have been previously reported in sucrose-fed rats; intracellular Ca^2+^ mishandling was proposed as the underlying mechanism (Carvajal and Baños, 2002; Vasanji et al., 2006; Okatan et al., 2016). Indeed, we consistently found a significant reduction in the Ca^2+^ transient amplitude of MetS cardiomyocytes (Figure 1B); our data agreed with previous reports (Hintz et al., 2003; Balderas-Villalobos et al., 2013; Okatan et al., 2016). Contrary to what might have been expected, we found no rate-dependent increase in the Ca^2+^ transient peak as previously reported (Bassani et al., 1995). The reason for this discrepancy is not apparent to us. Possible factors influencing this discrepancy are the different age and strain of our rats vs., the ones used by Bassani and cols. With regard to contraction, in the case of rodents (like rats and mice) the force-frequency relationship (FFR) is even or negative at very low *ex vivo* frequencies (Janssen and Periasamy, 2007). In our hands, the contraction-frequency relationship showed a pronounced drop in MetS cells while remaining unchanged in controls (Figure 1C). Of note, there is a dissociation between the important decrease in the amplitude of Ca^2+^ transients with increasing frequency and the less important or even lack of decrease in the amount of shortening. This dissociation may reveal an increase in Ca^2+^ myofilament sensitivity with increasing stimulation frequency, which appears as more important in control than in MetS cells. A frequency-dependent ‘sensitization’ of the myofilaments has been previously suggested (see for instance Gao et al., 1998). In addition, a possible explanation for the impairment of cell contractility in MetS cells might be related to the finding that in male *Wistar* rats under high-sucrose diet, both myosin and actomyosin Ca^2+^-ATPase are downregulated in the heart (Pierce et al., 1989); this should be addressed in the future.

In light of the fact that *k* SERCA was reduced in MetS cardiomyocytes, the observation that the Ca^2+^ transient decay time was not considerably augmented might seem surprising. However, it should be pointed out that additional Ca^2+^ clearing mechanisms are involved in the Ca^2+^ transient decline (i.e., Ca^2+^ extrusion by the NCX, cytoplasmic Ca^2+^-buffering proteins, dynamic RyR open time, etc.). Some of these are excluded when *k* SERCA is calculated as reported by Bode et al. (2011), unveiling a depressed SERCA function in MetS cells.

Because local, spontaneous Ca^2+^ release events (Ca^2+^ sparks) constitute the building blocks of Ca^2+^ transients (Cheng and Lederer, 2008), we studied the Ca^2+^ spark properties in MetS and control cells. Our results revealed a significant decrease in Ca^2+^ spark frequency and amplitude, reducing the diastolic Ca^2+^ leak in MetS cells (Figure 2); this suggests reduced RyR recruitment or activation (Hoang-Trong et al., 2015). Sucrose-fed rat hearts have a marked diminution of ATP and phosphocreatine content and creatine kinase activity (Carvajal et al., 2003). Because diastolic Ca^2+^ leak is energetically costly, the finding of a significant reduction of this parameter in MetS cardiomyocytes is not surprising, though it is in disagreement with other reports in insulin-resistant rats (Okatan et al., 2016; Sommese et al., 2016). However, when the heart is energetically compromised as in MetS condition, increasing the diastolic Ca^2+^ leak may add additional complications to already established energetic issues; thus, we propose that reducing the amount of Ca^2+^ leakage during diastole could be rather beneficial at rest, but may compromise heart function under high demand (i.e., β-adrenergic stimulus, stress, or exhausting exercise). Although an augmentation in diastolic [Ca^2+^]_i_ might participate in the impairment of the relaxation speed in MetS cells, previous reports in related insulin resistant models showed no changes in basal Ca^2+^ in cardiac cells (Dutta et al., 2001; Hintz et al., 2003), which is consistent with the decreased spark-mediated Ca^2+^ leak observed in our MetS model. However, it should be mentioned that increased diastolic Ca^2+^ levels have been also reported in sucrose- and fructose-fed rats (Okatan et al., 2016; Sommese et al., 2016). Moreover, at this moment we cannot rule out alterations in other forms of diastolic Ca^2+^ leak in MetS cardiomyocytes (Bovo et al., 2011).

In physiological conditions, free [Ca^2+^]_SR_ influences RyR2 activity by establishing the driving force for SR Ca^2+^ release (Györke and Terentyev, 2008). A reduction in the SR Ca^2+^ content induces a decrease in Ca^2+^ spark frequency (Satoh et al., 1997; Bers et al., 2003; Zima et al., 2010). In light of these findings, we analyzed caffeine-induced Ca^2+^ transients to assess whether the diminished diastolic Ca^2+^ leak observed in MetS cardiomyocytes was due to changes in SR Ca^2+^ load. Our results showed that MetS development does not modify the SR Ca^2+^ loading in cardiomyocytes, which is in agreement with some reports (Wold et al., 2005; Balderas-Villalobos et al., 2013) but not with others (Okatan et al., 2016; Sommese et al., 2016). The discrepancies are related to differences in the diastolic Ca^2+^ leak, which, in two of the abovementioned reports, was increased and explained the reduction in SR Ca^2+^ load (Okatan et al., 2016; Sommese et al., 2016). SERCA pump is one of the most studied proteins in the context of cardiomyopathies associated with diabetes; in the case of prediabetes, it is generally demonstrated that its activity decreases (Wold et al., 2005; Vasanji et al., 2006; Balderas-Villalobos et al., 2013; Okatan et al., 2016). Independently of the unchanged SR Ca^2+^ load, we corroborated the existence of a dysfunctional SERCA pump in MetS hearts, by the use of direct (SR Ca^2+^ uptake assays) and indirect (by calculating *k* SERCA) approaches (Figure 3). One conceivable explanation for the decrease in SERCA pump activity despite unchanged protein expression is that the oxidative stress level could be exacerbated in insulin resistant cardiomyocytes (Dutta et al., 2001; Hintz et al., 2003), this would increase the number of oxidized thiols in the SERCA pump, thereby depressing its activity (Balderas-Villalobos et al., 2013). Another explanation relies on the fact of reported reduced phosphorylation levels of PLN. In this work we found that pThr-17 PLN was significantly reduced, in agreement with one study in sucrose-fed rats reporting decreased phosphorylation of PLN at both Ser-16 and Thr-17, which could account for its reduced activity (Vasanji et al., 2006). On the other hand, SERCA pump activity is highly dependent on energy supply; thus, it is not surprising to find its activity depressed in MetS cardiomyocytes (Figure 3).

Neither SR Ca^2+^ load nor RyR2 protein expression was modified in MetS condition; therefore, this parameter cannot explain the depressed *in situ* activity of RyR2 in MetS cardiomyocytes. We performed [^3^H]-ryanodine binding assays to gain more insight into the molecular modifications of RyR2. Our results revealed that in MetS heart, the maximum number of functional RyRs was significantly reduced, though no significant changes (besides *Bmax* value) in Ca^2+^ dependent RyR activation/inactivation curves were revealed. We therefore examined RyR2 phosphorylation status at Serines 2808 and 2814 (in human and mouse nomenclature). These post-translational modifications, induced by kinases such as PKA and CaMKII, have been defined as a critical regulatory mechanism for this intracellular Ca^2+^ channel (Takasago et al., 1989, 1991; Huke and Bers, 2008; Camors and Valdivia, 2014). In our experimental model of MetS, we found a significant reduction in the phosphorylation level of Ser2814, which no doubt contributed to the impaired functional activity of RyRs in MetS hearts.

A seminal study in a dog model of MetS reported dysfunctional cardiac RyRs, characterized by a decreased capacity for [^3^H]-Ryanodine binding despite higher levels of Ser2808 phosphorylation compared with control hearts (Dincer et al., 2006). In our MetS model, the phosphorylation of Ser2808 remained unchanged. Perplexing a different study reported diminished phosphorylation of Ser2808 in MetS heart preparations, but did not further explore the impact of this variation in RyR functional activity (Paulino et al., 2010). Finally, additional studies have proposed that the “leaky” RyR2 phenotype in MetS cardiomyocytes is associated with augmented RyR phosphorylation at either Ser-2808 (Okatan et al., 2016) or Ser-2814 (Sommese et al., 2016). The only point where most publications—including ours—agree is about the fact that RyR2 protein expression remains unmodified in MetS models (Dincer et al., 2006; Paulino et al., 2010; Okatan et al., 2016; Sommese et al., 2016).

We are aware of the controversial role of Ser2808 phosphorylation in the abnormal activity of RyR2 in the setting of HF. The unsolved controversy is supported by a considerable amount of data that is primarily in favor of phasing out the importance of Ser2808 as the key player in HF (Valdivia, 2012); our data from a completely distinct cardiac disease support this idea. A definitive role for RyR2 phosphorylation has not yet been reached (Camors and Valdivia, 2014). Thus it is plausible that, in the case of MetS cardiomyopathy, a RyR2 that is dephosphorylated at a crucial residue (e.g., Ser2814) could be more difficult to activate. Indeed, the work of Dhindwal et al. (2017) supports the latter. This group proposed that phosphorylation causes RyR2 to adopt a protein conformation that requires less energy for the transition to the open state, using a structural basis to explain activating effects of phosphorylation in RyRs. Thus, we propose that in a metabolic condition such as MetS, the RyR is primarily dephosphorylated at Ser2814, thus impairing its physiological activation.

The downregulation of the CaMKII kinase could account for the reduced RyR phosphorylation, particularly at the Ser2814 site (Figure 5C). To test the involvement of this kinase, we evaluated the phosphorylation of PLN at Thr-17, which was significantly reduced in MetS heart preparations. However, CaMKII expression was not significantly changed (Supplementary Figure 2). It is important to note that CaMKII activity is regulated through several mechanisms. For example, CaMKII is susceptible to several post-translational modifications, including autophosphorylation, oxidation, S-nitrosylation, and O-GlcNAcylation, all of which are capable of sustaining the kinase’s activation (Mollova et al., 2015). Thus, we intend to address this issue in future work. Another mechanism that could be responsible for the apparent CaMKII downregulation could relate to disturbances of the CaM-CaMKII interaction due to the altered CaM oxidation state. However, this has not been demonstrated in the cardiac isoform (Robison et al., 2007).

## Conclusion

Principal findings of this work are that abnormal Ca^2+^ transient amplitude, contractile dysfunction; and impaired relaxation of MetS cardiomyocytes underlies intrinsic dysfunctional RyR2 and SERCA pump. Abnormal activity of RyRs was evidenced by its decreased ability to bind [^3^H]-ryanodine. Although the MetS condition does not modify RyR2 protein expression, its phosphorylation at Ser2814 is decreased, which impairs its capacity for activation during ECC. The dysfunctional RyRs, together with a decreased activity of SERCA pump due to decreased Thr17-PLN phosphorylation suggest a downregulation of CaMKII in MetS hearts, though its expression remained unchanged. Dysfunctional RyR2 and SERCA pump participate in the Ca^2+^ mishandling of MetS cardiomyocytes (i.e., reduced Ca^2+^ transient amplitude, and decreased Ca^2+^ spark-mediated Ca^2+^ leak), may account for the poor overall cardiac outcome reported in MetS patients and could be targeted for future therapies.

## Ethics Statement

This study was carried out in accordance with the ethical guidelines of the Mexican Official Norm (NOM-062-ZOO-1999) and the National Institutes of Health Guide for the Care and Use of Laboratory Animals (NIH publication updated in 2011); and was approved by the Institutional Bioethical Committee for Care and Handling of Laboratory Animals at the CINVESTAV-IPN (approved CICUAL Protocol No. 0105-14).

## Author Contributions

GF-M, TR-G, and TB-L contributed to the generation and characterization of the experimental model of MetS in rats, isolation of cardiomyocytes, confocal imaging of Ca^2+^ sparks, electrically stimulated Ca^2+^ transients, determination of SR Ca^2+^ load; [^3^H]-ryanodine bindings, Ca^2+^ uptake assays and data analysis. M-MM contributed towards SDS-PAGE, Western Blots, and densitometric analysis. AR contributed towards the experimental design, data analysis, data interpretation, and manuscript preparation.

## Conflict of Interest Statement

The authors declare that the research was conducted in the absence of any commercial or financial relationships that could be construed as a potential conflict of interest.

## Footnotes

1 http://maxchelator.stanford.edu/index.html
