# Supplementary material for: Impaired Activity of Ryanodine Receptors Contributes to Calcium Mishandling in Cardiomyocytes of Metabolic Syndrome Rats
Source: Front Physiol. 2019 Apr 30;10:520. doi: 10.3389/fphys.2019.00520 (PMC6503767; doi:10.3389/fphys.2019.00520)
Supplement: Supplementary file 1 [file Data_Sheet_1.docx]

**SUPPLEMENTARY DATA**

**
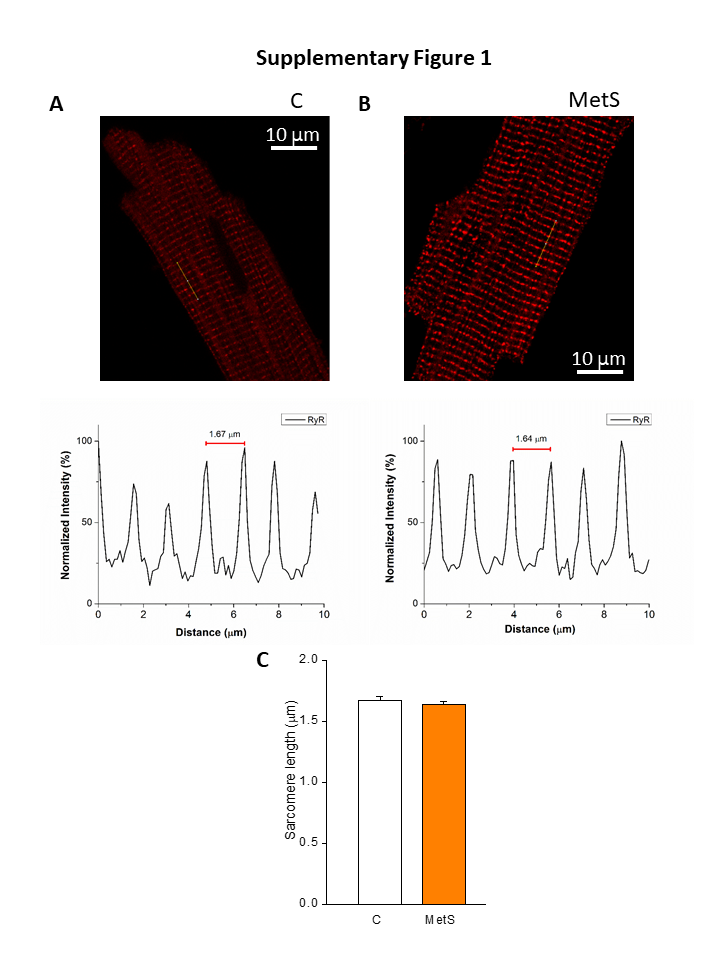
Supplementary Figure 1. Sarcomere length of Control and MetS cardiomyocytes.** Immunofluorescence images from control **(A)** and MetS **(B)** quiescent cardiomyocytes showing the fluorescence intensity distribution of RyRs (red signal) across several T-tubules (yellow line), with their corresponding intensity-distance plot. (See supplementary materials and methods). **(C)** Bar graph of average sarcomere length in RyR-immunostained cardiomyocytes from Control (C, N=5, *white bar*) and metabolic syndrome (MetS, N=5, *orange bar*) rats. All values in graphs are presented as M±SEM.


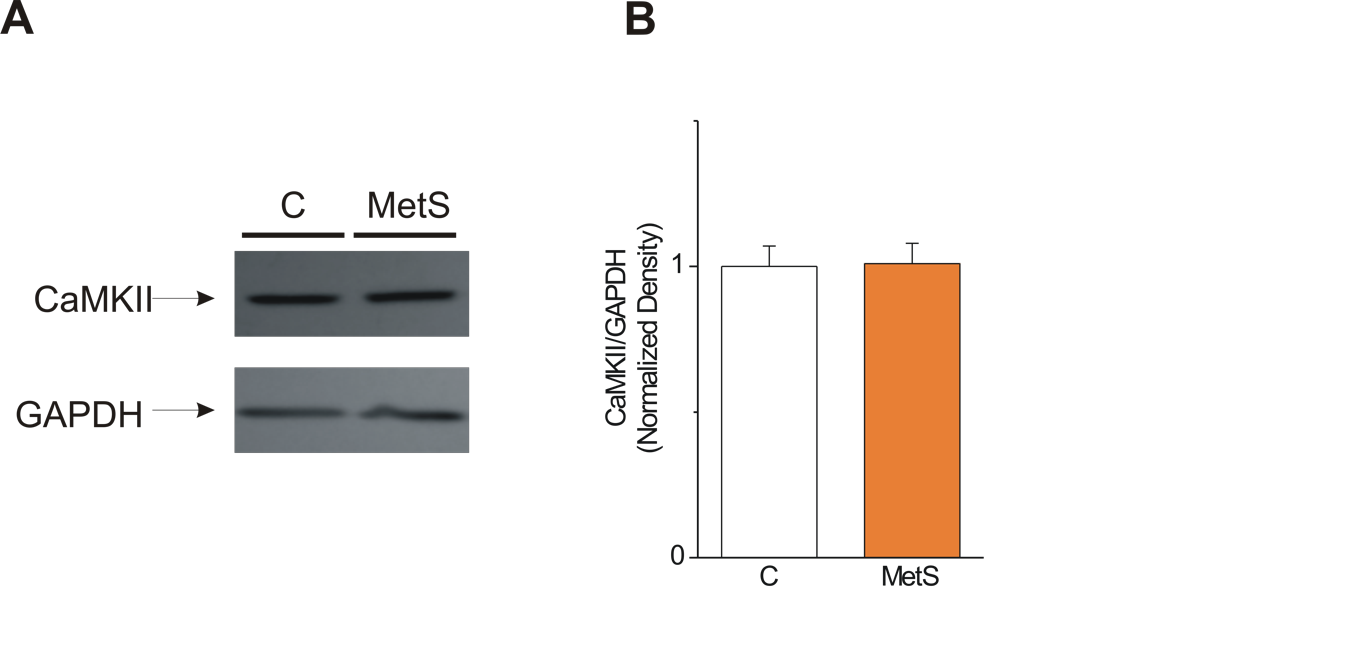


**Supplementary Figure 2. Cardiac CaMKII expression is unaffected in MetS condition.**

**(A)** Representative Western Blot images of total CaMKII in whole heart homogenates of Control (C) and metabolic syndrome (MetS) rats. GAPDH signals were used as loading controls. **(B)** Bar graph of normalized CaMKII expression levels of Control (C, N=7, *white bar*) and metabolic syndrome (MetS, N=8, *orange bar*) rats. All values in graphs are presented as M±SEM.

**Supplementary Materials and Methods**

*Cardiomyocyte immunostaining*

Determination of sarcomere length was performed indirectly by RyR immunostaining accordingly to a reported protocol (Rueda et al., 2006) with some modifications, as indicated below. Quiescent cardiomyocytes from MetS and control animals were diluted (1:10) in recording solution and allowed to adhere on laminin-coated (1:70 laminin:PBS) coverslips for at least 3 h at room temperature. Cells were fixed on the coverslips by immersion in fixative (4% paraformaldehyde, 2% picric acid in 0.1M PBS, pH 7.4) at room temperature for 10 min and subsequently washed 5X in PBS. After 10–20 min, cells were permeabilized in a solution of 0.2% Triton (in 1X PBS) and 5% normal goat serum (NGS, to block non-specific binding. Cat # PCN5000, GIBCO®, Thermo Fisher Scientific, Waltham, MA USA) for 30 min. Cells were incubated with anti-RyR2 antibody (C3-33, dil: 1:500) in PBS containing 1% NGS, for 2 h at room temperature and washed 5X, 5 min each, with PBS containing Triton X-100 (0.2%). After washing, cells were exposed for 1 h at room temperature to the secondary antibody (goat anti-mouse Rhodamine Red-X, 1:200 dilution, Cat # R-6393, Thermo Fisher Scientific, Waltham, MA USA), and again washed 5X 5min each, with PBS. Myocyte-containing coverslips were mounted onto glass slides using mounting solution (ProLong™ Gold Antifade Mountant, Cat # P36964, Thermo Fisher Scientific, Waltham, MA USA). Control staining (*not shown*) was done as described above with the exception that the anti-RyR2 was not added. Images were acquired using a Leica confocal microscope (SP5, Leica Microsystems, Wetzlar, Germany) using an oil-immersion X63 objective, by exciting Rhodamine Red-X at 570 nm and collecting the fluorescence at 590 nm. Sarcomere length analysis was performed by calculating the fluorescence profile plot considering at least six sarcomeres for each cell, using Image J (v. 1.50i). Fluorescence intensity was normalized respect to F_0_ value and reported respect to distance in $\mu$m. Confocal pixel size was of 0.12 x 0.12 x 0.8 µm for all images.

Rueda, A., Song, M., Toro, L., Stefani, E., and Valdivia, H. H. (2006). Sorcin modulation of Ca^2+^ sparks in rat vascular smooth muscle cells. *J. Physiol.* 576. doi:10.1113/jphysiol.2006.113951.
